# Supplementary material for: Potentially unsafe doses of local anesthetics in axillary brachial plexus block: A single-center retrospective cohort study
Source: PLoS One. 2026 Mar 11;21(3):e0344668. doi: 10.1371/journal.pone.0344668 (PMC12978474; doi:10.1371/journal.pone.0344668)
Supplement: S1 Checklist — (PDF) [file pone.0344668.s001.pdf]

STROBE Statement—checklist of items that should be included in reports of observational studies

	Item No.	Recommendation	Page No.	Relevant text from manuscript
**Title and abstract**	1	(*a*) Indicate the study's design with a commonly used term in the title or the abstract	1	"Potentially Unsafe Doses of Local Anesthetics in Axillary Brachial Plexus Block: a Single-Center Retrospective Cohort Study"
		(*b*) Provide in the abstract an informative and balanced summary of what was done and what was found	2	The abstract starts with: "Local anesthetic systemic toxicity is a rare but potentially life-threatening complication of regional anesthesia that can occur when high doses of local anesthetics are administered. This study aimed to evaluate the frequency of local anesthetic doses exceeding safe thresholds in axillary brachial plexus blocks using four different calculation methods.."
**Introduction**				
Background/rationale	2	Explain the scientific background and rationale for the investigation being reported	2-4	The introduction starts with: "Regional anesthesia, particularly peripheral nerve blocks, has become integral to modern anesthetic practice, offering effective pain control while reducing opioid requirements [1]. Axillary

				brachial plexus block is one of the most commonly performed procedures, valued for its reliability and ease of learning [2]. This technique involves injecting local anesthetics (LAs) near the brachial plexus to provide anesthesia for upper limb surgery.<br>Local anesthetic systemic toxicity (LAST) is a rare but potentially life-threatening complication that can occur when LAs enter systemic circulation in sufficient quantities . LAST manifests through symptoms ranging from mild neurological signs such as perioral numbness to severe complications including seizures and cardiovascular collapse [1]. While ultrasound guidance has improved safety by reducing vascular puncture rates, LAST continues to occur with reported incidence rates varying from 0.04% to nearly 1% depending on the clinical setting and diagnostic criteria [1,3–5]."
Objectives	3	State specific objectives, including any prespecified hypotheses	3,4	"The primary aim of this study was to evaluate the frequency of

				LA doses exceeding calculated safe thresholds in axillary brachial plexus blocks, using four different calculation methodologies. Secondary aims were to identify factors associated with potentially unsafe dosing and assess relationships between calculated dose exceedances and clinical manifestations of LAST.<br>The hypothesis was that a significant proportion of patients would receive doses exceeding recommended limits according to various calculation methods, with proportions varying substantially depending on the calculation approach used, thereby demonstrating the need for standardized dosing protocols in regional anesthesia practice.”
**Methods**				
Study design	4	Present key elements of study design early in the paper	4	“This was a retrospective cohort study carried out and reported according to the Strengthening the Reporting of Observational Studies in Epidemiology (STROBE) statement. “

Setting	5	Describe the setting, locations, and relevant dates, including periods of recruitment, exposure, follow-up, and data collection	4-9	For example: "The computerized files of all patients aged 18 or older who had undergone axillary brachial plexus block between January 2017 and December 2021 at Geneva University Hospitals (HUG) were included in this study. Files belonging to patients who had refused the reuse of their medical data and incomplete files i.e., those which did not contain enough data to calculate the primary outcome (weight, height, details of LA use), were excluded." Or "An electronic case report form (eCRF) was created using a REDCap electronic data capture tool hosted at HUG. All automatically extracted data were then imported, and all files were then reviewed to manually insert relevant data which had not or could not be automatically extracted. This included a review of intraoperative and follow-up notes to identify the presence of potential LAST symptoms."
Participants	6	(*a*) *Cohort study*—Give the eligibility criteria, and the sources and methods of selection of participants. Describe methods of follow-up	7,8	"The computerized files of all patients aged 18 or older who

		Case-control study—Give the eligibility criteria, and the sources and methods of case ascertainment and control selection. Give the rationale for the choice of cases and controls<br>*Cross-sectional study*—Give the eligibility criteria, and the sources and methods of selection of participants	had undergone axillary brachial plexus block between January 2017 and December 2021 at Geneva University Hospitals (HUG) were included in this study. Files belonging to patients who had refused the reuse of their medical data and incomplete files i.e., those which did not contain enough data to calculate the primary outcome (weight, height, details of LA use), were excluded. A general consent form for data reuse has been in place at our institution since 2017. However, as a sufficient number of records was required, and because data, once coded, could not lead to patient identification and posed no potential harm, we received authorization from the ethics committee to reuse data even without a signed general con-sent form. Files were also excluded when LAs other than lidocaine, levobupivacaine or ropivacaine were administered, when unusual concentrations were used (i.e., concentrations other than 0.25%, 0.375%, or 0.5% for levobupivacaine and

				ropivacaine, and other than 0.5% or 1% for lidocaine), or if more than 2 LAs had been given. Patients whose height was lower than 153 cm, thus preventing IBW calculation according to Devine's formula, were also excluded [12]. Additionally, patients were excluded if they underwent general anesthesia, if only a single nerve belonging to the axillary brachial plexus was blocked, or if other types of regional anesthesia were used. "
		(*b*) *Cohort study*—For matched studies, give matching criteria and number of exposed and unexposed<br>*Case-control study*—For matched studies, give matching criteria and the number of controls per case	N/A	This was not a matched study.
Variables	7	Clearly define all outcomes, exposures, predictors, potential confounders, and effect modifiers. Give diagnostic criteria, if applicable	8	"The primary outcome was the proportion of patients who received LA doses higher than the maximum safe dose according to most conservative, full set of calculation rules described above.<br>Secondary outcomes were the proportion of patients who received LA doses higher than calculated according to package inserts' recommendations, to AW and to IBW; the mean

				difference, in mg, between the doses administered and maximum safe doses ac-cording to package inserts' recommendations, AW, IBW and the full calculation procedure, when an overdose was considered present according to these criteria; the incidence of symptoms compatible with LAST; the incidence of vascular puncture; and the rate of neurostimulation and ultrasound use. The influence of patient age and sex, and of operator sex and experience, were also analyzed."
Data sources/ measurement	8*	For each variable of interest, give sources of data and details of methods of assessment (measurement). Describe comparability of assessment methods if there is more than one group	8,9	"An electronic case report form (eCRF) was created using a REDCap electronic data capture tool hosted at HUG. All automatically extracted data were then imported, and all files were then reviewed to manually insert relevant data which had not or could not be automatically extracted. This included a review of intraoperative and follow-up notes to identify the presence of potential LAST symptoms. We

				classified symptoms based on established criteria in the literature, which categorize manifestations as mild-to-moderate (perioral numbness, metallic taste, confusion, muscle twitching, etc.) or severe (seizures, loss of consciousness, respiratory depression, cardiac arrhythmias, severe hypotension, and cardiac arrest) [1].”
Bias	9	Describe any efforts to address potential sources of bias	N/A	There was no way to avoid the biases acknowledged (in the limitations – see below) given the design of this study.
Study size	10	Explain how the study size was arrived at	5	“It was estimated that, to detect a 1% dose difference with high precision (0.002) and 95% power, a sample of 2234 axillary brachial plexus blocks procedures would be necessary.”

Quantitative variables	11	Explain how quantitative variables were handled in the analyses. If applicable, describe which groupings were chosen and why	10	Among other things: “For weight-based analyses, the maximum safe dose of the first LA used was determined based on the patient’s AW, IBW, and finally according to the most conservative set of calculation rules described above.

				This maximum safe dose was considered equal to 100% of each of these calculated doses. The proportion of patients who had received an "overdose" (i.e., a dose over 100% of the calculated dose) and the related 95% CI were then reported accordingly. Additionally, when subgroups contained fewer than 5 patients, doses exceeding the maximum safe dose (calculated according to standard guidelines) were reported as median (Q1:Q3) in mg rather than mean values."
Statistical methods	12	(*a*) Describe all statistical methods, including those used to control for confounding	10	For example. "The Chi-squared test was used to search for an overdose difference between patients who had received a single LA and those who had received a mixture.<br>The influence of patient sex, patient age, operator experience and operator sex were assessed through multivariable logistic regression [26]. The effect size of each of these variables was reported through 95% CIs. The variables were selected according to their clinical relevance and to data availability. There was no risk of overfitting given the high overdose rates. Multicollinearity was ruled out using Spearman's test, and log-

				linearity was checked graphically. Goodness-of-fit was assessed using the Akaike Information Criterion (AIC).”
		(*b*) Describe any methods used to examine subgroups and interactions	10	“Multicollinearity was ruled out using Spearman’s test, and log-linearity was checked graphically. Goodness-of-fit was assessed using the Akaike Information Criterion (AIC).”
		(*c*) Explain how missing data were addressed	10	“No imputation methods were used for missing data. Analyses were performed only on available data (complete case analysis), and the extent of missing data for each variable is reported. P values < 0.05 were considered significant.”
		(*d*) *Cohort study*—If applicable, explain how loss to follow-up was addressed<br>*Case-control study*—If applicable, explain how matching of cases and controls was addressed<br>*Cross-sectional study*—If applicable, describe analytical methods taking account of sampling strategy	N/A	Not applicable (“single-shot” intervention).
		(*e*) Describe any sensitivity analyses	N/A	No sensitivity analysis was carried out in this study.
**Results**				
Participants	13*	(a) Report numbers of individuals at each stage of study—eg numbers potentially eligible, examined for eligibility, confirmed eligible, included in the study, completing follow-up, and analysed	N/A	Not applicable (“single-shot” intervention).
		(b) Give reasons for non-participation at each stage	N/A	Not applicable (“single-shot” intervention).
		(c) Consider use of a flow diagram	11	Cf. Figure 1
Descriptive data	14*	(a) Give characteristics of study participants (eg demographic, clinical, social) and information on exposures and potential confounders	11,12	Cf. Table 1

		(b) Indicate number of participants with missing data for each variable of interest	15	For example: "Vascular puncture was reported in 28 of 2387 analyzable cases (1.17%). Eight records did not contain enough information to be included in this analysis."
		(c) *Cohort study*—Summarise follow-up time (eg, average and total amount)	N/A	Not applicable ("single-shot" intervention).
Outcome data	15*	*Cohort study*—Report numbers of outcome events or summary measures over time	N/A	Not applicable ("single-shot" intervention).
		Case-control study—Report numbers in each exposure category, or summary measures of exposure		
		Cross-sectional study—Report numbers of outcome events or summary measures		
Main results	16	(*a*) Give unadjusted estimates and, if applicable, confounder-adjusted estimates and their precision (eg, 95% confidence interval). Make clear which confounders were adjusted for and why they were included	14	"The probability of potentially unsafe LA dose was not influenced by operator experience (Table 2). When the full calculation rules were considered, female patients and older patients were more likely to receive potentially unsafe LA dose, and female anesthesiologists were more likely to administer a potentially unsafe dose.<br>The association of patient and operator sex with the probability of potentially unsafe dose were consistent and of similar magnitude when IBW or AW were considered. The association with older age was still present when IBW was considered but disappeared when AW was used (Table 2)."

	(*b*) Report category boundaries when continuous variables were categorized	N/A	Apart from LA doses, which led to categorization as safe dose/overdose (see methods), no categorization was used.
	(*c*) If relevant, consider translating estimates of relative risk into absolute risk for a meaningful time period	N/A	Not applicable to this study.

Continued on next page

Other analyses	17	Report other analyses done—eg analyses of subgroups and interactions, and sensitivity analyses	12,13	Analyses according to actual weight and ideal body weight are reported.
**Discussion**				
Key results	18	Summarise key results with reference to study objectives	16	The discussion starts with "This retrospective cohort study demonstrates substantial variation in local anesthetic dosing practices for axillary brachial plexus blocks, with the proportion of potentially unsafe doses ranging from 29.5% to 64.8% depending on the calculation criteria applied."
Limitations	19	Discuss limitations of the study, taking into account sources of potential bias or imprecision. Discuss both direction and magnitude of any potential bias	19	Limitations are acknowledged and discussed. For example: "This study has several important limitations that must be considered when interpreting the results. The primary limitation relates to the absence of universally accepted rules for computing maximum safe local anesthetic doses. The four calculation methods used, while comprehensive, represent different philosophical approaches to dose determination rather than validated safety thresholds. The conservative consensus-based rules, in particular, may be overly restrictive and could overestimate the frequency of potentially problematic dosing. Our use of Devine's formula for ideal

				body weight calculation, while standard in our institution, represents another limitation as different weight calculation methods might yield different results. The single-center design limits the generalizability of findings, as dosing practices and patient populations may vary across different institutions and healthcare systems. The retrospective design increases the risk of bias, and selection bias cannot be ruled out since many files were excluded due to incomplete data or use of non-standard local anesthetic concentrations.”
Interpretation	20	Give a cautious overall interpretation of results considering objectives, limitations, multiplicity of analyses, results from similar studies, and other relevant evidence	18	Clinical implications are discussed. For instance: “The substantial variation in rates of potentially unsafe doses across the four methods (29.5% to 64.8%) illustrates how different safety criteria can dramatically alter the perception of dosing practices. This variation may explain some of the inconsistencies reported in the literature regarding local anesthetic dosing safety. The progressive increase from package insert recommendations to conservative consensus-based rules demonstrates

				the impact of incorporating patient-specific factors and conservative safety margins. Importantly, none of these calculation methods can be considered a definitive "gold standard," and the clinical relevance of each approach requires further validation.”
Generalisability	21	Discuss the generalisability (external validity) of the study results	19	The limitation of the single-centre and retroscpective design is acknowledged: “The single-center design limits the generalizability of findings, as dosing practices and patient populations may vary across different institutions and healthcare systems. The retrospective design increases the risk of bias, and selection bias cannot be ruled out since many files were excluded due to incomplete data or use of non-standard local anesthetic concentrations.”
**Other information**				
Funding	22	Give the source of funding and the role of the funders for the present study and, if applicable, for the original study on which the present article is based	N/A	This study was not funded.

*Give information separately for cases and controls in case-control studies and, if applicable, for exposed and unexposed groups in cohort and cross-sectional studies.

Note: An Explanation and Elaboration article discusses each checklist item and gives methodological background and published examples of transparent reporting. The STROBE checklist is best used in conjunction with this article (freely available on the Web sites of PLoS Medicine at http://www.plosmedicine.org/, Annals of Internal Medicine at http://www.annals.org/, and Epidemiology at http://www.epidem.com/). Information on the STROBE Initiative is available at www.strobe-statement.org.
