# Supplementary material for: Potentially unsafe doses of local anesthetics in axillary brachial plexus block: A single-center retrospective cohort study
Source: PLoS One. 2026 Mar 11;21(3):e0344668. doi: 10.1371/journal.pone.0344668 (PMC12978474; doi:10.1371/journal.pone.0344668)
Supplement: Attachment — Submitted filename: Response_editor.docx [file pone.0344668.s002.docx]

**Mélanie SUPPAN, M.D.**
Division of Anesthesiology,
Geneva University Hospitals,
1211 Geneva, Switzerland
+41223723311; [melanie.suppan@hug.ch](mailto:melanie.suppan@hug.ch)

October 14, 2025

PLOS ONE Editorial Office

plosone@plos.org

**Manuscript PONE-D-25-55020:** "Potentially unsafe doses of local anesthetics in axillary brachial plexus block: a single-center retrospective cohort study"

Dear Editor,

Thank you for your email regarding our manuscript submission. We have addressed both points raised and are pleased to resubmit our revised manuscript.

**Point 1: Human Participants Research Checklist**

We have completed the Human Participants Research Checklist and are uploading it as file type "Other" with this resubmission.

**Point 2: Participant Consent Details**

We have added detailed information regarding participant consent in the Methods section as follows:

Study design section (lines 91-93): We added an explicit statement that "the requirement for individual informed consent was waived by the ethics committee given the retrospective nature of this study using anonymized medical records." The manuscript already contained information about the general consent form system in place at our institution since 2017 and the ethics committee's authorization for data reuse.

Data collection and availability section (lines 167-168, 177-179, 181-182): We specified that data were accessed between August 2022 and December 2023 following ethics approval. We clarified that authors initially had access to potentially identifying information during data collection, but all data were subsequently anonymized and coded before analysis. The final dataset contains no personally identifiable information.

All modifications are visible in the tracked-changes version of the manuscript.

Documents Submitted:

Revised manuscript with tracked changes

Revised manuscript clean version

Completed Human Participants Research Checklist (file type "Other")

English translation of ethics committee approval (file type "Other")

Original ethics committee approval (file type "Other")

This response letter (file type "Other")

We trust these revisions adequately address your requirements. Thank you for your guidance.

Yours sincerely,

Dr. Mélanie Suppan

Corresponding Author

On behalf of all co-authors
