# Supplementary material for: Potentially unsafe doses of local anesthetics in axillary brachial plexus block: A single-center retrospective cohort study
Source: PLoS One. 2026 Mar 11;21(3):e0344668. doi: 10.1371/journal.pone.0344668 (PMC12978474; doi:10.1371/journal.pone.0344668)
Supplement: Attachment — Submitted filename: Response to Reviewers.docx [file pone.0344668.s003.docx]

**Editorial comments:**

**Comment 1.** Please ensure that your manuscript meets PLOS ONE's style requirements, including those for file naming. The PLOS ONE style templates can be found at

<https://journals.plos.org/plosone/s/file?id=wjVg/PLOSOne_formatting_sample_main_body.pdf> and
[https://journals.plos.org/plosone/s/file?id=ba62/PLOSOne_formatting_sample_title_authors_affiliations.pdf](https://track.editorialmanager.com/CL0/https:%2F%2Fjournals.plos.org%2Fplosone%2Fs%2Ffile%3Fid=ba62%2FPLOSOne_formatting_sample_title_authors_affiliations.pdf/1/010f019bbfcefdbc-dfbba44d-b51b-4b30-be4c-a4e1f5c7454d-000000/RgXsCu_nTT5ASjtRtTAibvPQ2f0SisP-l5Q3sS1d4-0=246)

**Response:** We have carefully reviewed PLOS ONE's style requirements and believe our manuscript complies with the journal's formatting guidelines, including file naming conventions. Should any specific elements require adjustment, we would be happy to make the necessary changes.

**Comment 2.** Please provide additional details regarding participant consent. In the ethics statement in the Methods and online submission information, please ensure that you have specified what type you obtained (for instance, written or verbal, and if verbal, how it was documented and witnessed). If your study included minors, state whether you obtained consent from parents or guardians. If the need for consent was waived by the ethics committee, please include this information.

**Response:** We thank you for this request and apologize for the lack of clarity regarding participant consent. In our original manuscript, the information regarding consent was dispersed across two sections of the Methods (Study design and Inclusion and exclusion criteria), which may have caused confusion.

To clarify:

- Our study only included adult patients (aged 18 years or older); no minors were included.
- A written general consent form for data reuse has been in place at our institution (Geneva University Hospitals) since 2017.
- However, given the retrospective nature of this study using anonymized medical records that posed no potential harm to participants, the regional ethics committee (Commission Cantonale d'Ethique de la Recherche, Geneva, Switzerland) waived the requirement for individual informed consent and authorized data reuse even for patients who had not signed the general consent form.

We have now consolidated and clarified this information in the Study design section of the Methods as follows: “*Approval by the regional ethics committee (Commission Cantonale d'Ethique de la Recherche CCER - Req 2022-01195, Geneva, Switzerland, Chairperson Prof B. Hirschel) was obtained on 16/08/2022.* *Only adult patients (aged 18 or older) were included in this study. A written general consent form for data reuse has been in place at our institution since 2017. However, given the retrospective nature of this study using anonymized medical records that posed no potential harm to participants, the ethics committee waived the requirement for individual informed consent and authorized data reuse even without a signed general consent form.*”

The corresponding information has been removed from the Inclusion and exclusion criteria section to avoid redundancy.

**Comment 3.** Thank you for stating the following in the Competing Interests section:

[I have read the journal's policy and the authors of this manuscript have the following competing interests: all authors were involved in the development of the LoAD Calc application, which was developed for research purposes only. This application is not monetized and will not be monetized in the future. The calculation rules from this application were used to define the most conservative calculations described in this manuscript.].

Please confirm that this does not alter your adherence to all PLOS ONE policies on sharing data and materials, by including the following statement: "This does not alter our adherence to PLOS ONE policies on sharing data and materials.” (as detailed online in our guide for authors <http://journals.plos.org/plosone/s/competing-interests>).  If there are restrictions on sharing of data and/or materials, please state these. Please note that we cannot proceed with consideration of your article until this information has been declared.

Please include your updated Competing Interests statement in your cover letter; we will change the online submission form on your behalf.

**Response:** We confirm that our competing interests do not alter our adherence to all PLOS ONE policies on sharing data and materials. There are no restrictions on sharing of data or materials. We have updated our Competing Interests statement as follows: “*All authors were involved in the development of the LoAD Calc application, which was developed for research purposes only. This application is not monetized and will not be monetized in the future. The calculation rules from this application were used to define the most conservative calculations described in this manuscript. This does not alter our adherence to PLOS ONE policies on sharing data and materials.*”

This updated statement has been included in our cover letter.

**Comment 4.** Please note that your Data Availability Statement is currently missing the repository name. If your manuscript is accepted for publication, you will be asked to provide these details on a very short timeline. We therefore suggest that you provide this information now, though we will not hold up the peer review process if you are unable.

**Response:** We thank you for pointing this out. Our anonymized dataset has been deposited in the Yareta repository, which is the institutional research data repository of the University of Geneva. The complete Data Availability Statement is as follows: “The anonymized dataset containing all data of the patients included in the analysis is available in the Yareta repository (University of Geneva) at: https://doi.org/10.26037/YARETA:3HCQXRX5ZFGULNWRCIBUCJGZIU”

**Reviewers' comments:**

**Reviewer #1:**

**Comment 1:** The article has been very nicely written with all data and study methodology being done in an appropriate matter. The language is simple yet precisely written. The article covers the area of a widely known subject regarding the use of anesthetics and its potential adverse effects seen throughout.

**Response:** We are delighted by your positive feedback and sincerely thank you for your kind words regarding our manuscript. It is very rewarding to know that you found our methodology appropriate and our writing clear and accessible. We truly appreciate the time and effort you dedicated to reviewing our work.

**Reviewer #2:**

**Comment 1:** Overall, the manuscript presents a well-conducted retrospective cohort study on potentially unsafe local anesthetic doses in axillary brachial plexus blocks. The use of four progressively conservative dosing methods is a particular strength and adds significant rigor.
I have the following minor comments for improvement:

**Response:** We sincerely thank you for your positive evaluation of our manuscript and for recognizing the rigor of our methodology, particularly the use of four progressively conservative dosing methods. We appreciate the time and effort you invested in providing constructive feedback. Below, we address each of the points you raised and detail the corresponding revisions we have made to strengthen the manuscript.

**Comment 2:** Sample size calculation: The study assumes detection of a 1% dose difference with 0.0022 precision. Clarify how this translates to the primary outcome (proportion of unsafe doses across methods).

**Response:** Thank you for this comment. Indeed, power analyses are rather uncommon in retrospective studies, and our aim was to ensure that we would be able to include enough patients to detect significant differences provided they existed. We therefore used the formula for sample size calculation in prevalence studies as described by Pourhoseingholi et al. (PMID: 24834239, reference n° 20). The formula proposed in this article is: n = Z^2^P(1-P)/d^2^, where n is the sample size, Z the confidence level, P the expected prevalence and d the precision – in this case, we used P/5 to compute it). Thus, the calculation was performed to detect a 1% difference with a precision of 0.002 and 95% power (n = 0.95^2^*0.01*(1-0.01)/0.002^2^ = 2233.6875, rounded to 2234), which makes the results particularly robust. To clarify this aspect, the following sentence was added to the methods section: “*To avoid fragility, precision was increased by dividing the estimated prevalence (0.01) by 5.*”

**Comment 3:** Table 1: Specify whether continuous variables are reported as mean ± SD or median (IQR).

**Response:** We thank you for this observation. Continuous variables are reported as mean ± SD, as stated in the Statistical Analysis section. We have added “±SD” where appropriate in Table 1. Furthermore, your comment made us realize that the “n, %” legends were sometimes misplaced or even missing, and therefore enabled us to correct this oversight.

**Comment 4:** Comorbidities/treatments: In the Table 1 legend, list the most common treatments altering LA metabolism (e.g., CYP inhibitors, heart failure meds).

**Response:** The following point was added to the legend: “*^5^Major CYP1A2 inhibitors such as ciprofloxacin, norfloxacin, and fluvoxamine, and major CYP3A inhibitors, such as azole antifungals, macrolides, calcium channel blockers, HIV antiretroviral therapy, and tyrosine kinase inhibitors*.”

**Comment 5:** Figure 2: Shows unsafe dose proportions for single agents (most conservative method). Consider adding similar panels for mixtures and other methods, with p-values, to enhance visual comparison.

**Response:** Thank you for this suggestion. We carefully considered adding similar panels for mixtures and other calculation methods. However, we decided against this for two reasons. First, the vast majority of mixtures (97.6%) consisted of ropivacaine with lidocaine, which would limit meaningful visual comparison across mixture types. Second, we were concerned that adding multiple panels might overwhelm the reader and detract from the main message. The detailed breakdown of potentially unsafe doses for mixtures and across all calculation methods is provided in the text and in Table 2. Should you or the Editor feel that additional figures would enhance the manuscript, we would be happy to reconsider.

**Comment 6:** Multivariate logistic regression: Only 4 variables were included. Justify exclusion of others (e.g., weight, comorbidities, interacting drugs)—were they tested in univariate analysis or omitted for clinical/logical reasons?

**Response:** You are right, this is an important question. The four variables included (patient age, patient sex, operator sex, operator experience) were selected a priori based on clinical relevance and data availability. Weight was not included as an independent predictor because it is already incorporated into the outcome calculation itself (potentially unsafe dose is weight-dependent), which would introduce collinearity. Similarly, comorbidities and interacting drugs are embedded in the most conservative calculation method and were therefore not analyzed as separate predictors for the same reason. We have clarified this in the Methods section: “*The variables were selected according to their clinical relevance and to data availability. Weight, comorbidities, and interacting drugs were not included as independent predictors because they are already incorporated into the outcome calculation, which would introduce collinearity.*”

**Comment 7:** LAST symptom identification: Elaborate on how LAST symptoms were extracted and classified from records (e.g., keyword search, clinical criteria, inter-rater agreement).

**Response:** We thank you for this comment. Most of this information is already provided in the Methods section. All files were manually reviewed to search for LAST symptoms, as stated: “*This included a review of intraoperative and follow-up notes to identify the presence of potential LAST symptoms.*” Classification was carried out according to established criteria in the literature, with symptoms categorized as “*mild-to-moderate (perioral numbness, metallic taste, confusion, muscle twitching, etc.) or severe (seizures, loss of consciousness, respiratory depression, cardiac arrhythmias, severe hypotension, and cardiac arrest).*” To further clarify, we have slightly revised the wording to specify that files were *“manually reviewed”*.

**Comment 8:** Power for LAST association: With only 19 LAST events (0.79%), the analysis may be underpowered. Acknowledge this limitation or provide a post-hoc power calculation.

**Response:** We absolutely agree with you on this account. The following limitation has been added as advised: “*Finally, given the limited incidence of LAST symptoms, no definitive conclusion could be drawn regarding an association between the use of potentially unsafe LA doses and the occurrence of LAST.*”

**Comment 9:** These minor clarifications will strengthen an already robust and clinically relevant manuscript

**Response:** We thank you once again for your time and comments and believe that our manuscript has indeed been enhanced by virtue of the review process.

**Reviewer #3:**

**Comment 1:** Thank you for the opportunity to review this manuscript. I found the topic highly relevant, especially because safe local anesthetic dosing is a challenge in everyday decision making. The authors have done an impressive job assembling a large dataset and applying multiple dosing frameworks. The manuscript is clear and well-organized. Still, there are several areas where more explanation would help readers fully understand the reasoning behind the methods and the implications of the findings.

**Response:** We sincerely thank you for your thoughtful evaluation and for recognizing the clinical relevance of our work. We are grateful for the time you dedicated to providing detailed and constructive feedback. Below, we address each of your comments and describe the revisions we have made to improve the manuscript.

**Comment 2:** One of the main strengths of this study is that it compares LA doses using four different calculation methods, from the simplest (package-insert values) to the most conservative individualized approach. However, the manuscript does not fully explain why these four specific criteria were selected. A brief explanation of how each method reflects real-world practice, why they differ, and what the authors hoped to learn from comparing them would really help orient readers. Even two or three sentences explaining the clinical relevance, why these methods matter to anesthesiologists making dosing decisions, would make this section much more accessible.

**Response:** We thank you for this valuable suggestion. We agree that explaining the rationale behind our choice of four calculation methods would help orient readers. We have added the following paragraph to the Methods section:

“*Four different calculation methods were selected to represent a spectrum of approaches encountered in clinical practice, from the simplest to the most individualized. These range from basic package insert recommendations, commonly used as a default reference, to actual weight-based calculations reflecting routine clinical practice, to ideal body weight-based calculations addressing pharmacokinetic concerns in overweight and obese patients, and finally to conservative consensus-based rules incorporating patient-specific factors known to affect LA metabolism (age, organ dysfunction, drug interactions) that are rarely systematically considered in everyday practice. Comparing these progressively more conservative approaches allows clinicians to benchmark their own institutional practices against multiple standards and illustrates how the assessment of dosing safety varies depending on the criteria applied.*”

**Comment 3:** I also think the manuscript would benefit from a short discussion about the rationale for mixing LAs. This is a common practice in some institutions but not in others, and there is still debate about whether mixing offers meaningful clinical advantages. In the methods, the authors apply the principle that systemic toxicity is additive, which is reasonable, but readers may wonder whether mixing agents should even be encouraged or if it introduces unnecessary complexity. Explaining the pharmacological reasoning and briefly mentioning what the literature says about the benefits or risks of mixing would be more relevant. This does not need to be long, just enough to show that the authors have considered why mixtures occur and what is known about their safety profile.

**Response:** We agree that discussing the rationale for LA mixtures and the variability of this practice would strengthen the manuscript. We have distributed this information across three sections to provide appropriate context while maintaining methodological focus.

In the Introduction, we have expanded the existing sentence on mixtures to reflect the variability of this practice: “*The proximity to major vessels, combined with the need for multiple injections, increases risks of intravascular injection and systemic absorption. Furthermore, the practice of mixing LA to combine the rapid onset of one agent with the prolonged duration of another, while common in some institutions, remains variable and debated, with limited evidence supporting superior clinical outcomes compared to single agents. When used, such mixtures further complicate dose calculations.*” Along with the following reference: Nestor CC, Ng C, Sepulveda P, Irwin MG. Pharmacological and clinical implications of local anaesthetic mixtures: a narrative review. Anaesthesia. 2022 Mar;77(3):339-350. doi: 10.1111/anae.15641. Epub 2021 Dec 14. PMID: 34904711.

In the Methods section, we have adapted the paragraph on mixture calculation : “*Since systemic toxicity of LA is considered additive when multiple agents are used, proportional dose adjustments were applied for mixtures. The maximum safe dose of the first LA was calculated. If this dose already exceeded safety thresholds, it was considered potentially unsafe regardless of additional agents. If the first LA dose was within safe limits, the percentage of the maximum safe dose already utilized was determined. The remaining “safety margin” was then applied proportionally to the second agent. For example, if a patient received 70% of their calculated maximum safe dose of ropivacaine, only 30% of the maximum safe dose of lidocaine could be administered without potentially exceeding cumulative toxicity thresholds.*”

In the Discussion section, we have modified the paragraph on mixtures to expand on the clinical implications: “*Both single LA agents and mixtures of different LA led to potentially unsafe doses, with mixtures consistently accounting for the highest proportion of potentially unsafe doses regardless of the calculation method used. This suggests that the practice of mixing LA, while common and intended to combine favorable pharmacological properties such as rapid onset with extended duration, substantially increases the likelihood of exceeding calculated safety thresholds. Furthermore, the calculation rules for determining maximum doses of LA when using mixtures lack standardization. The clinical evidence supporting enhanced efficacy of LA mixtures remains limited, and the pharmacokinetic advantages may be offset by the increased complexity of dose calculations. Given these considerations, institutions that routinely use LA mixtures may benefit from implementing standardized protocols or decision-support tools to minimize the risk of inadvertent overdosing.*”

**Comment 4:** Statistical analysis is straightforward, but the use of AIC needs a bit more explanation. AIC is perfectly acceptable for comparing models, but readers may not immediately understand why it was chosen here or how it helps interpret the results. A short justification makes this clearer. Similarly, since the study looks at factors like patient age, patient sex, operator experience, and operator sex, it would be helpful to say whether the authors explored any potential interactions. In a setting like this, certain variables may influence each other, and even a brief statement acknowledging whether interactions were tested or why they were not would improve confidence in the model.

**Response:** You are perfectly right, the AIC is infrequently used, but we believe that it allowed us to assess which dose calculation approach best captured the relationships between patient/operator characteristics and potentially unsafe dosing. To help the reader understand its use, the following sentence was added: “*The AIC was used because, since the weight-based calculation approaches used different outcome definitions rather than different predictor sets, they are non-nested and cannot be compared using likelihood ratio tests.”.* We did not include interaction terms in our primary analysis. Given the exploratory nature of this study and the number of potential interactions relative to outcome events, we prioritized a parsimonious model to avoid overfitting.

**Comment 5:** Overall, this manuscript tackles an important and practical question, and the dataset is strong. Addressing the points above would make the work even clearer and more useful for clinicians who rely on these dosing principles every day.

**Response:** We thank you for your constructive comments and encouragement. We are confident that addressing these points has enhanced the clarity and practical utility of our manuscript for clinicians.
