# Supplementary material for: Potentially unsafe doses of local anesthetics in axillary brachial plexus block: A single-center retrospective cohort study
Source: PLoS One. 2026 Mar 11;21(3):e0344668. doi: 10.1371/journal.pone.0344668 (PMC12978474; doi:10.1371/journal.pone.0344668)
Supplement: Attachment — Submitted filename: Response_to_Reviewers_auresp_2.docx [file pone.0344668.s004.docx]

Dear Editor,

We are grateful for the opportunity to revise our manuscript entitled “Potentially unsafe doses of local anesthetics in axillary brachial plexus block: a single-center retrospective cohort study” (Manuscript ID: PONE-D-25-55020R1) and would like to sincerely thank you and the reviewers for their time and thoughtful evaluation of our work. The comments provided have been valuable in helping us improve the clarity and overall quality of the manuscript. We have carefully considered each point raised and have revised the manuscript accordingly. We believe the revised version is significantly strengthened and hope it now meets the standards required for publication in Plos One.

A point-by-point response to each comment is provided below. All changes made to the manuscript are highlighted in the revised version.

Yours sincerely,

Mélanie Suppan, on behalf of all co-authors

**Reviewers' comments:**

**Reviewer #3:**

**Comment 1:** This is a well designed retrospective Cohort study that addresses an important and clinically relevant question. The manuscript is well written and structured, however, if few points are interpreted with clarity this strengthens the manuscript.

**Response:** Thank you for this positive assessment of our work. We have carefully addressed the specific points raised in the subsequent comments and believe these revisions strengthen the manuscript.

**Comment 2:** The finding that female patients and female anesthesiologists were more likely to be associated with potentially unsafe dosing is interesting but sensitive. Although the Discussion acknowledges possible unmeasured confounding (lines 347-355), this finding is presented prominently in the Abstract without sufficient qualification. The language in the Abstract should be softened to clearly state that these associations are observational and unexplained. In the Discussion, consider briefly expanding on potential structural or workflow-related explanations (e.g., differences in case mix, teaching roles, or patient allocation) to avoid overly individual-level interpretations.

**Response:** Thank you for raising this point. We agree that these associations are difficult to adequately qualify within the constraints of an abstract and have therefore removed this finding from the Abstract, where it could be misinterpreted. It remains presented with appropriate qualification in the Discussion, which we have expanded to include potential structural and workflow-level explanations as suggested.

The revised paragraph now reads: “*The finding that female anesthesiologists were more likely to administer potentially unsafe doses was unexpected and contrasts with established literature showing that female physicians are generally more risk-averse in procedural settings [34,35]. This counterintuitive finding requires careful interpretation and may reflect unmeasured confounders not captured in our retrospective analysis,* *such as differences in case allocation, patient mix, teaching responsibilities, or other workflow-related factors. Alternatively, it could suggest that gender-based differences in clinical practice may not uniformly apply across all domains of medical decision-making or may be influenced by institutional training patterns and protocols. The retrospective nature of our study prevented us from establishing causality, and this finding highlights the need for prospective research to confirm this association and explore potential underlying mechanisms.*”

**Comment 3:** Also, the manuscript uses multiple terms such as “potentially unsafe dose,” “dose exceeding recommendations,” and “overdose.” For clarity, consider defining one preferred term early and using it consistently throughout the manuscript.

**Response:** Thank you for this observation. We have adopted **“potentially unsafe dose”** as the preferred term throughout the manuscript, including in the Methods section, as it best reflects the nuanced, calculation-based nature of our findings without implying that clinical toxicity will necessarily occur. All instances of alternative terminology have been replaced accordingly.
